# A highly accurate quantum optimization algorithm for CT image reconstruction based on sinogram patterns

**DOI:** 10.1038/s41598-023-41700-6

**Published:** 2023-09-01

**Authors:** Kyungtaek Jun

**Affiliations:** Quantum Research Center, QTomo, Busan, South Korea

**Keywords:** Applied mathematics, Computational science, Quantum information

## Abstract

Computed tomography (CT) has been developed as a nondestructive technique for observing minute internal images in samples. It has been difficult to obtain photorealistic (clean or clear) CT images due to various unwanted artifacts generated during the CT scanning process, along with the limitations of back-projection algorithms. Recently, an iterative optimization algorithm has been developed that uses an entire sinogram to reduce errors caused by artifacts. In this paper, we introduce a new quantum algorithm for reconstructing CT images. This algorithm can be used with any type of light source as long as the projection is defined. Assuming an experimental sinogram produced by a Radon transform, to find the CT image of this sinogram, we express the CT image as a combination of qubits. After acquiring the Radon transform of the undetermined CT image, we combine the actual sinogram and the optimized qubits. The global energy optimization value used here can determine the value of qubits through a gate model quantum computer or quantum annealer. In particular, the new algorithm can also be used for cone-beam CT image reconstruction and for medical imaging.

## Introduction

Tomography is predominantly a nondestructive technology. Computed tomography (CT) is a technique that allows nondestructive internal observations of a given sample. CT has been widely used to observe internal structures in biology, archaeology, geoscience, and materials science^1–6^. In particular, CT is extensively used for medical diagnoses. Electron tomography (ET) is another technique utilized in several fields with different uses. CT is mainly used for samples of a few micrometers or more in size, but ET can be used for far smaller molecular structures (nanometers to angstroms). ET is also widely used to study three-dimensional internal structures in biology and materials science^7–10^.

There are three main types of tomographic systems: spiral CT^11^, electron tomography (ET)^12^, and synchrotron X-ray tomography^13^. Each CT system has been developed to suit the sizes and characteristics of various samples. The back-projection technique used in these tomographic systems can be largely attributed to three algorithms: the iteration algorithm^14^, fast Fourier transform algorithm^15^, or artificial intelligence algorithm^16^. In 2022, an optimization algorithm for the entire sinogram was introduced^17^. This algorithm iteratively uses an optimization method to reduce the difference between the sinogram generated by the Radon transformation and the sinogram obtained by the projection of the CT image. To reduce the error compared to the existing iterative algorithm, image preprocessing was performed to satisfy the Beer‒Lambert law as much as possible^18^. Since this algorithm uses the optimization algorithm for the entire sinogram, it has minimal errors with respect to artifacts that may partially occur^19–24^.

In this paper, we introduce a new quantum optimization algorithm that accurately obtains the real internal structure of a sample when two conditions are met: the experimental data are error-free, and the mathematical projection of the undetermined CT image to make a sinogram matches the X-ray projection of a real-life sample to an entire sinogram. The algorithm represents the pixels of a CT image in qubits. The algorithm uses projection on the undetermined CT image to create the undetermined sinogram. The original projection data used here use an experimentally obtained sinogram from a CT system. After that, the algorithm obtains a quadratic unconstrained binary optimization (QUBO) or Ising model through optimized calculations of the undetermined sinogram and the experimentally obtained sinogram. This model determines the value of all qubits by obtaining a global optimal energy from a gateway quantum computer or quantum annealer. Finally, the determined qubit combination related to the global optimal energy can represent the internal structure of a sample. Our quantum optimization algorithm for CT image reconstruction has three major advantages when certain conditions are met. The first advantage is that our algorithm can be used for CT images of any light source type. One condition for this advantage is that the algorithm can be used if the projection method used to obtain the experimental data is defined. A second advantage is that it can reconstruct highly accurate CT images, assuming that the projected data satisfy the Beer‒Lambert law and that there are enough clean data without errors. This also assumes that we have a sufficient number of logical qubits available and that the projection can be calculated mathematically. In this ideal case, our algorithm can find an exact value for the X-ray mass attenuation of the sample. The final advantage is that the new algorithm is highly resistant to artifacts in the projected images. Since our quantum optimization algorithm calculates the difference for the whole sinogram, it can perform accurate approximations even if an error appears in specific parts. In addition, since motion artifacts can be corrected in CT images, the calculations are easier and more accurate than previous methods for modifying projection images^21–24^. Historically, it has been difficult to reconstruct a clean CT image through back-projection algorithms that utilized cone-beam CT (CBCT) systems due to the geometric limitations of the light source (X-ray) used. Our new algorithm does not suffer from this issue. Furthermore, our optimization algorithm can approximate a clear image even if the number of projected images is not sufficient^17^. Therefore, we believe that our new quantum algorithm for CT image reconstruction will be useful in the field of medical imaging. In this paper, we use the Radon transform to test our new algorithm. Quantum optimization calculations are performed in D-Wave Advantage, D-Wave simulator, and IBM Quantum.

## Method

A quantum optimization model for linear systems was developed and implemented^25^. In this paper, we have applied this optimization model algorithm for application to CT image reconstruction^2^. A sinogram is an image created by accumulating projected images of an object according to the projection angle. We introduce QUBO and Ising models that can represent the entire sinogram. These models can reconstruct CT images with the lowest energy in quantum annealing. Additionally, this approach can be implemented through the quantum approximate optimization algorithm (QAOA)^26^ in a gate model quantum computer. The new optimization model can reconstruct a CT image utilizing a quantum computer, assuming that there are enough qubits. The new algorithm should use sinograms for the entire projected image in the CBCT system and use sinograms according to each axial level in the parallel-beam CT system. In this paper, we introduce a reconstruction method for one axial level to simplify the explanation. Additionally, we use a Radon transform for projection.

### Energy optimization algorithm for the Radon transform

**Figure 1:**
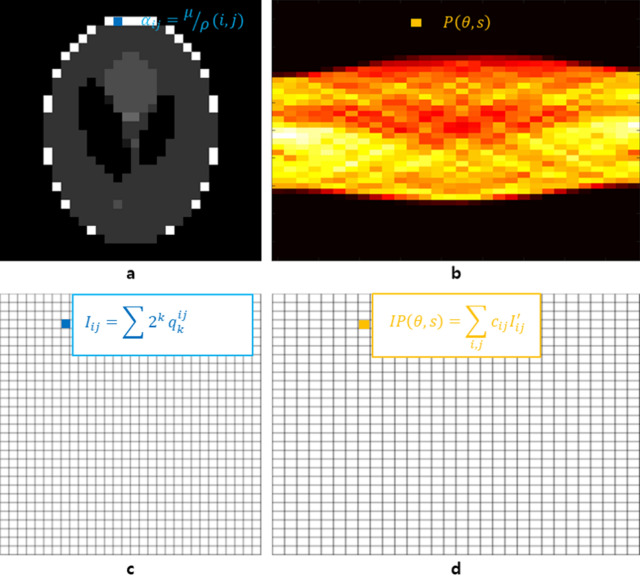
A sample, a CT image, and their two sinograms to illustrate the optimization algorithm. (**a**) This sample is a $30\times 30$ Shepp–Logan phantom image. In the case of a general three-dimensional sample, it represents a cross section of the sample corresponding to its axial level. (**b**) This sinogram was obtained by using the Radon transform with the number of pixels equal to the size of the sample in (**a**). When using the data obtained from the CT system, it corresponds to the sinogram of the X-ray image. (**c**) An undetermined CT image composed of combinations of logical qubits. (**d**) The sinogram obtained by applying the projection to (**c**) in the same way as to obtain the sinogram in (**b**). In this figure, the Radon transform was applied to (**c**).

Consider a space of size $n\times n$ that includes the cross section of a Shepp–Logan phantom. Let ${\alpha }_{ij}=\frac{\mu }{\rho }(i,j)$ be the X-ray mass attenuation coefficient^27^ and a natural number. We can assume this value as the number at the $(i,j)$ position of the sample (see Fig. 1a). Figure 1b shows a sinogram produced by the projections of this sample. The value of each pixel in the sinogram can be expressed as $P(\theta ,s)$. Now, we consider a reconstructed image $I$ of size $n\times n$ consisting of qubits that can be represented in Fig. 1a (see Fig. 1c). If the maximum of integer-valued pixels is less than ${2}^{m+1}$, then each pixel in the reconstructed image is represented by one of the combinations of qubits and binary numbers in Eq. 1.

1 $${I}_{ij}=\sum_{k=0}^{m}{{2}^{k}q}_{k}^{ij}$$

Here, ${q}_{k}^{ij}$ is $0$ or $1$, and ${I}_{ij}$ represents any integer from $0$ to ${2}^{m+1}-1$.

**Figure 2:**
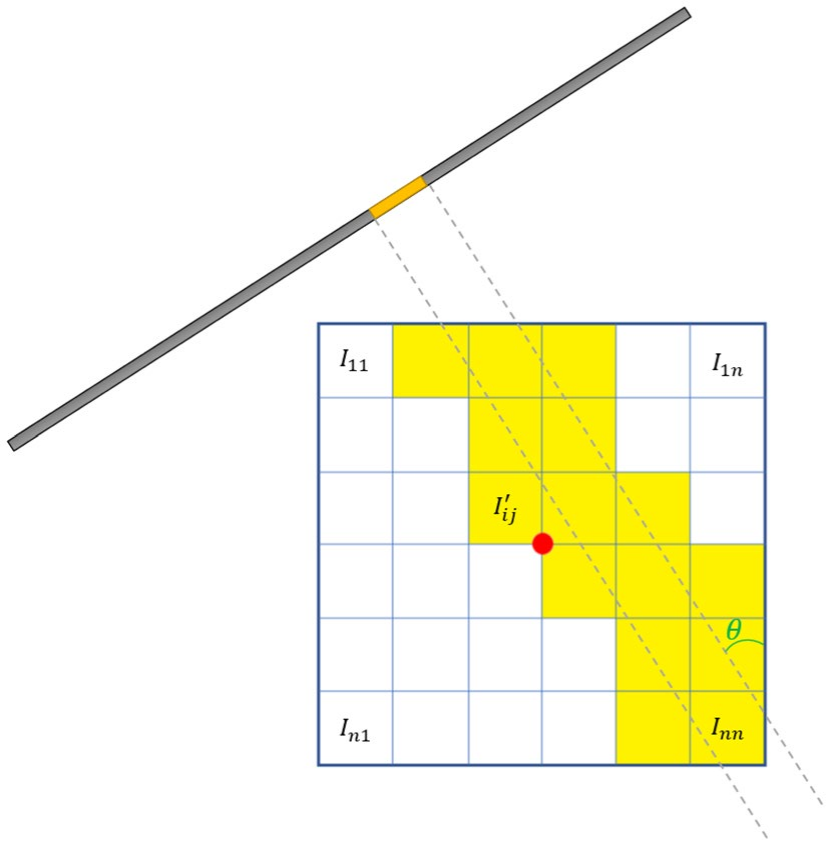
Illustration of the projection of a CT image with respect to the projection angle $\theta$. The $\left(i,j\right)$ pixel of the CT image is denoted by ${I}_{ij}$. Pixels ${I}_{ij}$ that affect $IP(\theta ,s)$ when projected with respect to the projection angle are referred to as ${I}_{ij}{\prime}$.

To apply the optimization algorithm to the experimental sinogram, we use a Radon transform on the undetermined CT image. Let $IP$ be the undetermined sinogram transformed by the CT image $I$. For the projection angle $\theta$, the s-th position of $IP$ is calculated as in Eq. 2.

2 $$IP\left(\theta ,s\right)=\sum_{i,j}{c}_{ij} {I}^{\prime}_{ij}$$

where ${I}^{\prime}_{ij}$ denotes the pixel that affects $IP\left(\theta ,s\right)$ when the CT image is projected and ${c}_{ij}$ is the overlapping area when ${I}^{\prime}_{ij}$ is projected (see Fig. 2). Using the square of the difference between $P\left(\theta ,s\right)$ and $IP\left(\theta ,s\right)$ (see Fig. 1b, d), the optimization model is calculated as follows:

3 $${\left(IP\left(\theta,s\right)-{P}\left(\theta,s\right)\right)}^{2}={\left(\sum_{{{i}},{{j}}}{{{c}}}_{{{i}}{{j}}}{{{I}}}_{{{i}}{{j}}}^{\boldsymbol{^{\prime}}}-{{P}}\left({{\theta}},{{s}}\right)\right)}^{2}$$

4 $$={\left(\sum_{{{i}},{{j}}}{{{c}}}_{{{i}}{{j}}}\sum_{{{k}}=0}^{{{m}}}{{2}^{{{k}}}{{q}}}_{{{k}}}^{{{i}}{{j}}}-{{P}}\left({{\theta}},{{s}}\right)\right)}^{2}$$

5 $$={\left(\sum_{{{i}},{{j}}}{{{c}}}_{{{i}}{{j}}}\sum_{{{k}}=0}^{{{m}}}{{2}^{{{k}}}{{q}}}_{{{k}}}^{{{i}}{{j}}}\right)}^{2}-2{{P}}\left({{\theta}},{{s}}\right)\sum_{{{i}},{{j}}}{{{c}}}_{{{i}}{{j}}}\sum_{{{k}}=0}^{{{m}}}{{2}^{{{k}}}{{q}}}_{{{k}}}^{{{i}}{{j}}}+{\left({{P}}\left({{\theta}},{{s}}\right)\right)}^{2}$$

In Eq. 5, the second term is a linear term in the QUBO model, and the third term represents a part of the optimization value. The first term is calculated as follows:

6 $$\begin{aligned} {\left(\sum_{i,j}{{{c}}}_{{{i}}{{j}}}\sum_{k=0}^{m}{{2}^{k}q}_{k}^{ij}\right)}^{2}=\sum_{i,j}{\left({{{c}}}_{{{i}}{{j}}}\sum_{k=0}^{m}{{2}^{k}q}_{k}^{ij}\right)}^{2} +2 \sum_{\begin{array} {c} i,{i}^{\prime},j,{j}^{\prime} \\ i\neq {i}^{\prime}\, or \,j\neq {j}^{\prime} \end{array} }{\left({{{c}}}_{{{i}}{{j}}}\sum_{k=0}^{m}{{2}^{k}q}_{k}^{ij}\right)}{\left({{{c}}}_{{{i}^{\prime}}{{j}^{\prime}}}\sum_{k^{\prime}=0}^{m}{{2}^{{k}^{\prime}}q}_{k^{\prime}}^{i^{\prime}j^{\prime}}\right)}\end{aligned}$$

7 $$\begin{aligned}={\sum_{i,j}}{\sum_{k=0}^{m}}{2}^{2k} c_{{ij}^{2}} {q}_{k}^{ij}+ \sum_{i,j} \sum_{0\le k<{k}^{\prime}\le m} {{2}^{k+{k}^{\prime}+1} c_{{ij}^{2}}q}_{k}^{ij}{q}_{k^{\prime}}^{{i}{j}}+\sum_{\begin{array} {c} i,{i}^{\prime},j,{j}^{\prime} \\ i\neq {i}^{\prime}\, or \,j\neq {j}^{\prime} \end{array} } \sum_{0\le k<{k}^{\prime}\le m} {{2}^{k+{k}^{\prime}+1} c_{ij} c_{i^{\prime}j^{\prime}}q}_{k}^{ij}{q}_{k^{\prime}}^{{i^{\prime}}{j^{\prime}}}\end{aligned}$$

To derive Eq. 7 from Eq. 6, we can convert the square terms by using ${\left({q}_{k}^{ij}\right)}^{2}={q}_{k}^{ij}$ because ${q}_{k}^{ij}$ is $0$ or $1$. In Eq. 7, $i,j$, and $k$ cannot be equal to ${i}^{\prime},{j}^{\prime}$ and ${k}^{\prime}$ at the same time. We can calculate the first term in Eq. 5 as the sum of linear and quadratic terms as in Eq. 7.

Now, we can compare two sinograms $P$ and $IP$. To compute the energy minimization model, we subtract the values for each pixel in the two sinograms and square them.

8 $$F(\theta ,s)=\sum_{\theta =0}^{180-d\theta }\sum_{s=1}^{n}{\left(\left(IP-P\right)\left(\theta ,s\right)\right)}^{2}$$

where $\theta$ is the projection angle, $s$ is the position of the sensor, and $d\theta$ is the amount of change in the projection angle. Now, $F(\theta ,s)$ is expressed in linear terms and quadratic terms excluding constant terms. In the QUBO model, constant terms are excluded. The minimum value of the QUBO model is the opposite sign of the summation of constant terms.

### Energy optimization algorithm for the real X-ray data

**Figure 3:**
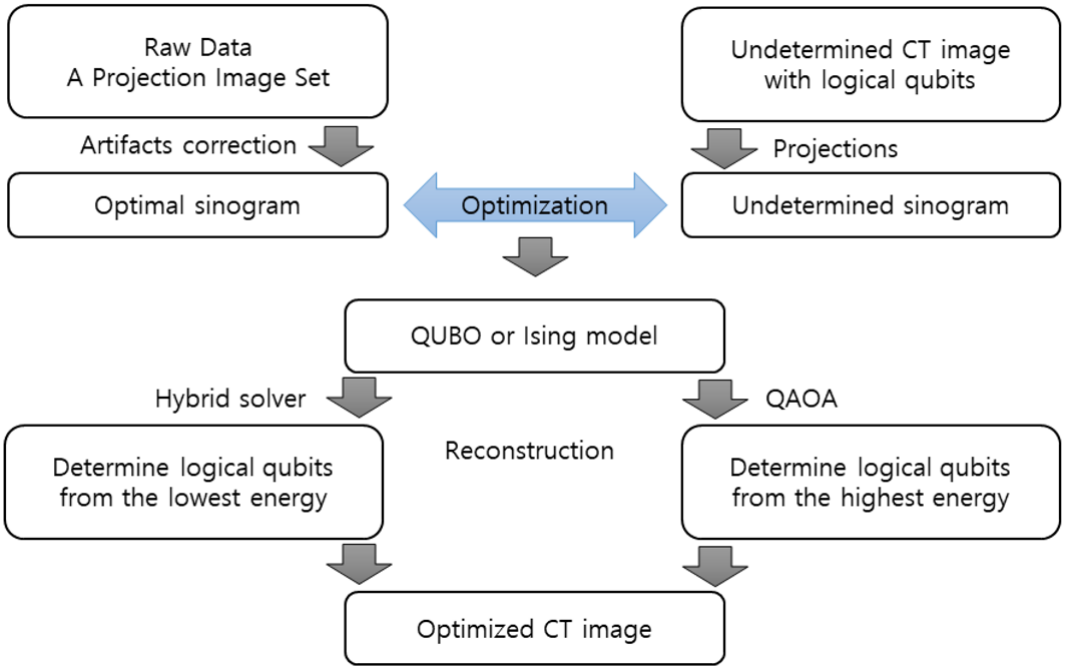
Flowchart of the process of obtaining a CT image. To reconstruct a CBCT image, the entire 3D sinogram is needed. To reconstruct a parallel-beam CT image, a sinogram along one axial level is needed. In this case, optimization must be applied to all axial levels to obtain 3D CT images.

Figure 3 shows a flowchart for reconstructing a CT image from X-ray data. To obtain a CT image optimized for the internal structure of a real sample, we calibrate the X-ray image. The mathematical projection including the Radon transform is directly proportional to the thickness of the sample's mass attenuation coefficient for each projected location. When the projection does not penetrate the sample, the value of the transmitted position appears as zero. On the other hand, the X-ray projection image has some differences from the projection due to various errors. We need mathematical adjustments to the X-ray image to reduce these errors. First, to represent the empty space, the average value of the empty space is subtracted from the entire X-ray image. The processed X-ray image shows the X-ray density. If the X-ray image has a positive or negative deviation, the overall image is modified to satisfy the Beer‒Lambert law to be effective in applying the new algorithm. Because the X-ray coherent effect is system-dependent, it helps to incorporate this effect into a mathematical projection. If the X-ray image contains high-density areas such as metal or pixels that create ring artifacts, it is recommended to remove these areas as well. From now on, the CT image can be reconstructed in a similar way to the one calculated above. First, we create an undetermined CT image in logical qubits. A mathematical projection, such as the X-rays used in the experiment, is applied to this CT image. We compute the quantum optimization model of the sinogram obtained from the experiment and the undetermined sinogram. As introduced in Fig. 3, the values of logical qubits are determined through the QAOA algorithm or quantum annealing. Finally, we can reconstruct an accurate CT image.

## Result and implementation

**Figure 4:**
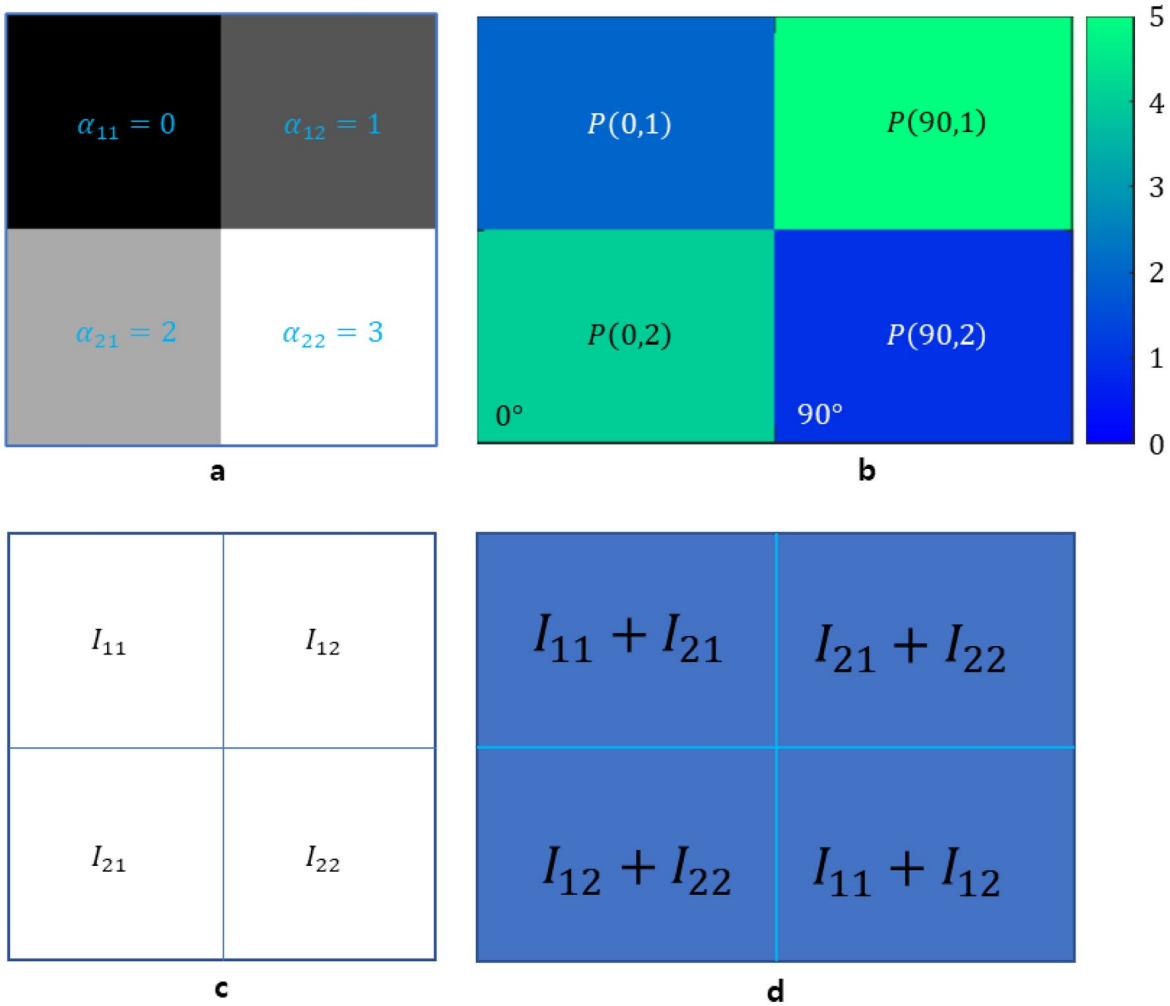
Example used in the QUBO model. (**a**) $2\times 2$ image sample with given mass attenuation coefficients. (**b**) A sinogram coming from the image sample in (**a**). (**c**) An undetermined CT image with qubit variables. (**d**) An undetermined sinogram with two projection angles of 0 and 90° by the Radon transform of the undetermined CT image in (**c**).

In this paper, we use the Radon transform for projection to show the results of the new algorithm. We use a $2\times 2$ image sample to formulate the QUBO model. Suppose we have an image sample as shown in Fig. 4a. To obtain an exact solution for a $2\times 2$ reconstruction image, a sinogram consisting of two projections is required (see Fig. 4). We use two qubits for each position. Therefore, ${I}_{ij}$ can be expressed as $\sum_{k=0}^{1}{{2}^{k}q}_{k}^{ij}$. A sinogram consists of projections with projection angles of 0 and 90 degrees. Now, let us formulate the QUBO model.

9 $${\left(\left(IP-P\right)\left(\mathrm{0,1}\right)\right)}^{2}={\left({{\sum }_{k=0}^{1}{{2}^{k}q}_{k}^{11}+{\sum }_{k=0}^{1}{{2}^{k}q}_{k}^{21}-\alpha_{11} }-{\alpha }_{21}\right)}^{2}$$

10 $$\begin{aligned} = & \left( {1 - 2\left( {\alpha _{{11}} + \alpha _{{21}} } \right)} \right)q_{0}^{{11}} + \left( {4 - 4\left( {\alpha _{{11}} + \alpha _{{21}} } \right)} \right)q_{1}^{{11}} + \left( {1 - 2\left( {\alpha _{{11}} + \alpha _{{21}} } \right)} \right)q_{1}^{{21}} \\ & + \left( {4 - 4\left( {\alpha _{{11}} + \alpha _{{21}} } \right)} \right)q_{1}^{{21}} + 2(2q_{0}^{{11}} q_{1}^{{11}} + q_{0}^{{11}} q_{0}^{{21}} + 2q_{0}^{{11}} q_{1}^{{21}} + {\text{}}2q_{1}^{{11}} q_{0}^{{21}} \\ & + 4q_{1}^{{11}} q_{1}^{{21}} + 2q_{0}^{{21}} q_{1}^{{21}} ) + \left( {\alpha _{{11}} + \alpha _{{21}} } \right)^{2} \\ \end{aligned}$$

When Eq. 10 is calculated from Eq. 9, ${\left({q}_{k}^{ij}\right)}^{2}={q}_{k}^{ij}$ is used. ${\left(\left(IP-P\right)\left(\mathrm{0,2}\right)\right)}^{2}$, ${\left(\left(IP-P\right)\left(\mathrm{90,1}\right)\right)}^{2}$, and ${\left(\left(IP-P\right)\left(\mathrm{90,2}\right)\right)}^{2}$ can be calculated in a similar way. The QUBO matrix $QM$ is obtained in Eq. 11.

11 $$QM=\left(\begin{array}{ccc}\begin{array}{ccc}-4 & 8& 2\\ 0& -4& 4\\ \begin{array}{c}0\\ 0\\ \begin{array}{c}0\\ \begin{array}{c}0\\ 0\\ 0\end{array}\end{array}\end{array}& \begin{array}{c}0\\ 0\\ \begin{array}{c}0\\ \begin{array}{c}0\\ 0\\ 0\end{array}\end{array}\end{array}& \begin{array}{c}-8\\ 0\\ \begin{array}{c}0\\ \begin{array}{c}0\\ 0\\ 0\end{array}\end{array}\end{array}\end{array}& \begin{array}{ccc}4& 2& 4\\ 8& 4& 8\\ \begin{array}{c}8\\ -12\\ \begin{array}{c}0\\ \begin{array}{c}0\\ 0\\ 0\end{array}\end{array}\end{array}& \begin{array}{c}0\\ 0\\ \begin{array}{c}-12\\ \begin{array}{c}0\\ 0\\ 0\end{array}\end{array}\end{array}& \begin{array}{c}0\\ 0\\ \begin{array}{c}8\\ \begin{array}{c}-20\\ 0\\ 0\end{array}\end{array}\end{array}\end{array}& \begin{array}{cc}0& 0\\ 0& 0\\ \begin{array}{c}2\\ 4\\ \begin{array}{c}2\\ \begin{array}{c}4\\ -16\\ 0\end{array}\end{array}\end{array}& \begin{array}{c}4\\ 8\\ \begin{array}{c}4\\ \begin{array}{c}8\\ 8\\ -28\end{array}\end{array}\end{array}\end{array}\end{array}\right)$$

The condition $F(\theta ,s)\ge 0$ produces the lowest energy $-{\left({\alpha }_{11}+{\alpha }_{21}\right)}^{2}-{\left({\alpha }_{12}+{\alpha }_{22}\right)}^{2}-{\left({\alpha }_{21}+{\alpha }_{22}\right)}^{2}-{\left({\alpha }_{11}+{\alpha }_{12}\right)}^{2}=-46$ for the QUBO model. The minimum energy in quantum annealing is -46 and is obtained by the following qubit vector:

12 $$\left({q}_{0}^{11}, {q}_{1}^{11},{q}_{0}^{12},{q}_{1}^{12},{q}_{0}^{21},{q}_{1}^{21},{q}_{0}^{22},{q}_{1}^{22}\right)=(0, 0, 1, 0, 0, 1, 1, 1)$$

In the D-Wave Advantage system, the occurrence having the lowest energy is 515 from 1000 annealings. As a result of quantum annealing, the reconstructed image composed of qubit variables in Fig. 4c has the same values as the sample in Fig. 4a.

The QUBO model and Ising model are mathematically equivalent. To convert the QUBO model to the Ising model for the minimum energy model, the following transformation is needed.

13 $${q}_{i}\to \frac{{\sigma }_{i}+1}{2}$$

For the QUBO matrix $QM$ in Eq. 11, the Ising matrix $IM$ in Eq. 14 is calculated by applying the transformation in Eq. 13. We obtained the Ising matrix using the ‘dimod.qubo_to_ising’ function provided by D-wave Ocean software.

14 $$IM=\left(\begin{array}{ccc}\begin{array}{ccc}3 & 2& 0.5\\ 0& 6& 1\\ \begin{array}{c}0\\ 0\\ \begin{array}{c}0\\ \begin{array}{c}0\\ 0\\ 0\end{array}\end{array}\end{array}& \begin{array}{c}0\\ 0\\ \begin{array}{c}0\\ \begin{array}{c}0\\ 0\\ 0\end{array}\end{array}\end{array}& \begin{array}{c}1\\ 0\\ \begin{array}{c}0\\ \begin{array}{c}0\\ 0\\ 0\end{array}\end{array}\end{array}\end{array}& \begin{array}{ccc}1& 0.5& 1\\ 2& 1& 2\\ \begin{array}{c}2\\ 2\\ \begin{array}{c}0\\ \begin{array}{c}0\\ 0\\ 0\end{array}\end{array}\end{array}& \begin{array}{c}0\\ 0\\ \begin{array}{c}-1\\ \begin{array}{c}0\\ 0\\ 0\end{array}\end{array}\end{array}& \begin{array}{c}0\\ 0\\ \begin{array}{c}2\\ \begin{array}{c}-2\\ 0\\ 0\end{array}\end{array}\end{array}\end{array}& \begin{array}{cc}0& 0\\ 0& 0\\ \begin{array}{c}0.5\\ 1\\ \begin{array}{c}0.5\\ \begin{array}{c}1\\ -3\\ 0\end{array}\end{array}\end{array}& \begin{array}{c}1\\ 2\\ \begin{array}{c}1\\ \begin{array}{c}2\\ 2\\ -6\end{array}\end{array}\end{array}\end{array}\end{array}\right)$$

The D-Wave quantum annealer can calculate the lowest energy for the QUBO model or Ising model directly. In the IBM quantum computer using the gate model, two models obtain the maximum energy value through QAOA provided by Qiskit. When converting from a QUBO matrix $QM$ to an Ising matrix $IM$, a constant term corresponding to a 1/4 * coefficient appears in each quadratic term. Therefore, the global minimum energy of $IM$ is − 20, and the global maximum energy of $-IM$ is 20. For the first sample in Fig. 4a, two optimal energies, − 20 and 20, were obtained with the simulators provided by the D-Wave system and IBM Quantum, respectively.

**Figure 5:**
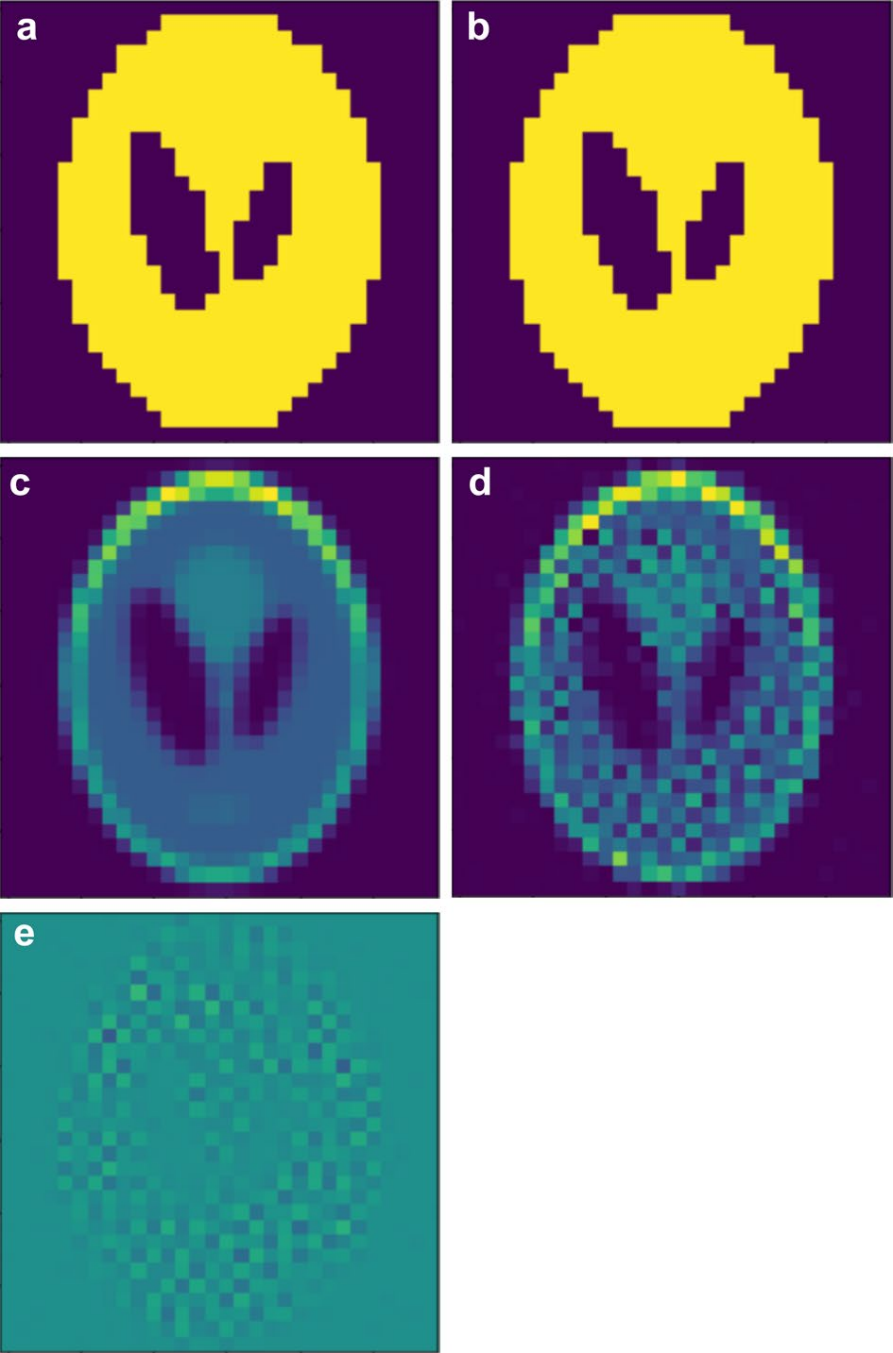
CT images reconstructed by applying the quantum optimization algorithm to a Shepp–Logan phantom. The size of the phantom used for testing is $30\times 30$. (**a**) This image is the binary Shepp–Logan phantom used for testing. (**b**) This image is a CT image reconstructed using a hybrid solver in the D-Wave system for (**a**). (**c**) This image is a Shepp–Logan phantom used in the test, and each pixel is rounded to have integer values from 0 to 1023. (**d**) The CT image for the sample in (**c**) is reconstructed using a hybrid solver for 3 s. (**e**) This image shows the difference between the original image in (**c**) and the CT image in (**d**). Each pixel darker than the pixels located outside the Shepp–Logan phantom is the case where the CT image has a higher pixel value, and the bright pixels are vice versa.

Our second test sample for a D-Wave hybrid solver is a 2D Shepp–Logan $30\times 30$ phantom. We used the binary Shepp–Logan phantom, as shown in Fig. 5a, to test the QUBO model for CT image reconstruction. The size and pixel values of the image used here are chosen so that the hybrid solver can find the overall minimum energy for the QUBO model at one iteration. We apply a Radon transform to this image to obtain projection data, which is an original sinogram. Since binary numbers can be represented by one qubit, each pixel of an undetermined CT image consists of one qubit. A Radon transform is applied to this CT image to create an undetermined sinogram. We calculate the QUBO model required for CT image reconstruction using the difference between the original sinogram and the undetermined sinogram. The CT image of Fig. 5b was reconstructed using the hybrid solver of the D-Wave system. To reconstruct a CT image that is identical to the original image, the minimum value for the QUBO model is $-\sum_{\theta =0}^{180-d\theta }\sum_{s=1}^{n}{(P(\theta ,s)}^{2})$. The global minimum energy for this test in the quantum annealer is $-\hspace{0.17em}\mathrm{225,518.918231688}$. We obtained the minimum energy of − $\mathrm{225,518.910675}$ using the hybrid solver of the D-Wave system. The CT image reconstructed with the value of qubits determined as the minimum energy is shown in Fig. 5b and is exactly the same as the original image. The Shepp–Logan phantom in Fig. 5c is a sample rounded up so that each pixel value is an integer from 0 to 1023. In this case, 10 qubits are required to represent each pixel. Similar to the above, if the minimum energy for this QUBO model is obtained using the hybrid solver, the CT image as shown in Fig. 5d can be reconstructed. The global lowest energy of the QUBO model we are targeting is − $33656657418.458885$, but in the quantum annealer, we obtain − $33653444028.65625$ as the minimum energy. Although the relative error of energy was less than 0.01%, there was a difference between the reconstructed CT image and the original image, as shown in Fig. 5c. Figure 5e shows the difference between the sample and the reconstructed CT image.

We tested the reconstruction of CT images according to the number of projections with the new algorithm in a D-Wave hybrid solver. We used the sinogram made with the Shepp–Logan phantom in Fig. 5a as the original data. The minimum energy expected in a quantum annealer is the negative sign of the sum of the squares of each pixel value in the sinogram. We obtained the lowest energy using a hybrid solver and reconstructed the CT image using qubits. The number of projections used in the sinogram was tested from 30 to 18. In Table 1, we can confirm that the CT reconstruction image is identical to the original image.

**Table 1:** CT images according to the number of projections used in the new quantum-optimized reconstruction algorithm.

Projection number	Expected the lowest energy	Result from hybrid solver	Relative error
30	− 225518.91823	− 225518.91068	0
27	− 203026.37744	− 203026.37890	0
24	− 180491.95504	− 180491.95515	0
21	− 157920.25285	− 157920.25185	0
18	− 135340.57831	− 135340.58023	0

In the table, the expected lowest energy represents the minimum energy that the QUBO model can have. The result from the hybrid solver is the lowest energy obtained by using the hybrid solver for the QUBO model. The relative error indicates the difference between the Shepp–Logan phantom and the CT reconstruction image.

**Figure 6:**
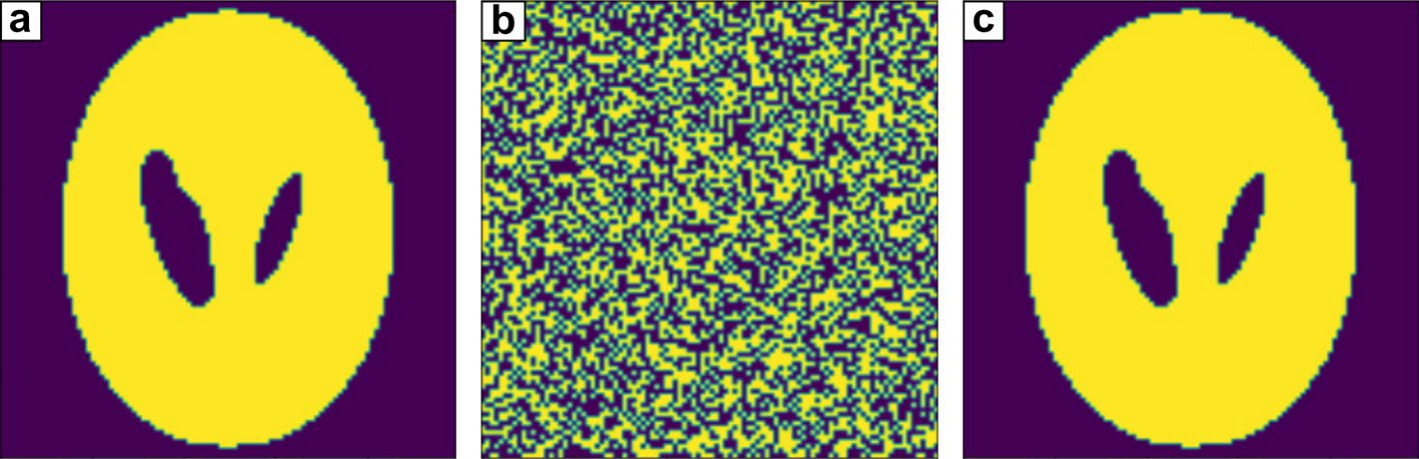
Performance comparison of the TABU solver and hybrid solver for CT image reconstruction. (**a**) This image is the $100\times 100$ 2D binary Shepp–Logan phantom used for testing. (**b**) CT image reconstructed using the TABU solver. (**c**) CT image reconstructed using the hybrid solver.

Finally, we compared the performance of D-Wave's hybrid solver and TABU solver using test samples. The sample used for testing is a binarized 2D Shepp–Logan phantom. For CT image reconstruction, the quantum computing usage time used for each calculation is 3 s. When using a $50\times 50$ image that required 2500 qubits, both the TABU solver and hybrid server reconstructed the same image as the test sample. When using a $100\times 100$ image that requires 10,000 qubits, as shown in Fig. 6a, the CT images obtained using the TABU solver and hybrid solver are shown in Fig. 6b, c, respectively. Theoretically, the global minimum energy required to reconstruct a CT image is − 32341735.63925959, but the TABU solver obtained an energy of − 27973314.080052175. The CT image reconstructed using the TABU solver had 5024 pixels with different values from the test sample. The hybrid solver obtained the global minimum energy, which matched the theoretical minimum energy to two decimal places. The reconstructed CT image was identical to the image sample and had no errors.

## Discussion

The National Lung Cancer Screening Trial recently demonstrated that lung cancer mortality can be reduced using low-dose computed tomography (LDCT)^28^. In addition, LDCT has recently been used for diagnosing breast cancer, which accounts for a high proportion of female cancer deaths^29, 30^. As the importance of LDCT systems increases, so does the importance of algorithms used for image reconstruction. Algorithms used for CT image reconstruction in the LDCT system can be largely divided into iterative-based^14, 31^ and AI-based algorithms^16^. They are all based on optimization. It is very difficult to find a solution in a short timeframe for an optimization problem with many variables. This is because optimization problems are heavily influenced by initial values, and most solutions find local solutions rather than global solutions. However, since the quantum optimization algorithm finds the global minimum/maximum energy for the QUBO/Ising model, it can reconstruct the most accurate image from the given data. Additionally, if the information of the projection data is further improved by the existing method, the reconstructed image will also become clearer. This can promote considerable progress in LDCT imaging.

Since our new algorithm uses an energy optimization model for the whole sinogram, it has two additional advantages over other algorithms. First, it is not significantly affected by noise and produces good results even if the number of X-ray images is not sufficient. A sinogram is obtained by X-ray transmissions while the sample is rotating. Even if a certain part of the X-ray image has errors, the new algorithm reduces the influence of those errors from other angles of the X-ray image. Second, our new algorithm is not confined by the number of $nx$ projections of an $nx\times nx$ sample, which typically limits CT image reconstruction. When a CT image has variables corresponding to ${nx}^{2}$ for an $nx\times nx$ sample, our new algorithm can reconstruct an accurate CT image with fewer than $nx$ projections. To obtain an accurate CT image using previously proposed algorithms, $nx$ X-ray images of different angles are needed. However, our new algorithm can overcome this limitation. Since this algorithm can reconstruct an accurate CT image using a small number of projections, the radiation received by the sample during CT scanning is reduced. This is one of the important factors for medical CT. We expect this algorithm to play a particularly important role in medical CT image reconstruction. We believe that quantum optimization algorithms will offer great advances in imaging diagnostics using CT images.

Basically, the QUBO model or Ising model can be represented by an upper triangular matrix or a symmetric matrix. The more nonzero numbers there are in the upper triangular matrix, the more logical qubits are used. In the case of the D-Wave advantage, approximately 180 fully connected logical variables are available^32^. This is a number that the quantum annealer can calculate when using one qubit variable for each pixel in a CT image of 13 by 13 pixels. Since the size of a commonly used CT image is $500\times 500$ or more, at least 250,000 logical qubits are needed. The hybrid solver of the D-Wave system provides up to one million variables and 200 million biases. The number of logical qubits used in Fig. 5d is 9000, showing a relative error of less than 0.01%. Despite the small error when obtaining the minimum energy, the reconstructed CT image still has a difference from the original. However, we believe that if the hybrid solver can obtain a more accurate minimum energy, it will be a huge step forward in medical CT imaging. We believe that a hybrid solver in which a quantum computer and a classic computer work together will change our lives in a shorter time because of the lack of connectivity between qubits in quantum computers. From our results, we believe that the hybrid solver is close to being used for CT image reconstruction. In addition, we will continue to develop mathematical algorithms that allow the QUBO model for CT image reconstruction to better find the global minimum energy.

Our new QUBO model is available in both quantum annealers and gated model quantum computers. However, our new algorithm seems more suitable for quantum annealers. The reason is that the circuit depth is too long to obtain results for the QUBO or Ising model in a gate model quantum computer through QAOA. For example, the IBM quantum computer has low connectivity between qubits, and the basic gate does not include an RZZ gate. The possibility of scheduling annealing cycles contributes to confirming that it is a valid approach for solving computationally complex problems^33–35^. In the results of Table 1, when 900 logical qubits are used in the CT image reconstruction, the theoretical minimum energy and the minimum energy of the quantum annealer coincide by about 8 digits, and noise occur less than 0.01. The uncertainty depends on the system of the quantum annealer and the time used to calculate the minimum energy, and is approximately $O({10}^{-2})$^36^.

### Supplementary Information

Supplementary Information.

## Data Availability

The Python code used in this paper is in the supplementary material. More detailed code can be found on the author's GitHub site (https://github.com/ktfriends/Quantum_CT_reconstruction).
