# Author Correction: A highly accurate quantum optimization algorithm for CT image reconstruction based on sinogram patterns

**DOI:** 10.1038/s41598-024-63404-1

**Published:** 2024-06-10

**Authors:** Kyungtaek Jun

**Affiliations:** Quantum Research Center, QTomo, Busan, South Korea

Correction to: *Scientific Reports* 10.1038/s41598-023-41700-6, published online 01 September 2023

The original version of this Article contained an error in the Energy optimization algorithm. As a result, the variable ‘C_ij_’ in Equations 2, to 7 and Figure 1 was incorrectly displayed.

In Equation 2,

$$ IP\left( {\theta ,s} \right) = c_{ij} \mathop \sum \limits_{i,j} I_{ij}^{{\prime }} $$

now reads:

$$IP\left(\theta ,s\right)=\sum_{i,j}{c}_{ij}{I}_{ij}^{\prime}$$

In Equation 3,

$${\left({\varvec{I}}{\varvec{P}}\left({\varvec{\theta}},{\varvec{s}}\right)-{\varvec{P}}\left({\varvec{\theta}},{\varvec{s}}\right)\right)}^{2}={\left({{\varvec{c}}}_{{\varvec{i}}{\varvec{j}}}\sum_{{\varvec{i}},{\varvec{j}}}{{\varvec{I}}}_{{\varvec{i}}{\varvec{j}}}^{{{\prime}}}-{\varvec{P}}\left({\varvec{\theta}},{\varvec{s}}\right)\right)}^{2}$$

now reads:

$${\left({\varvec{I}}{\varvec{P}}\left({\varvec{\theta}},{\varvec{s}}\right)-{\varvec{P}}\left({\varvec{\theta}},{\varvec{s}}\right)\right)}^{2}={\left(\sum_{{\varvec{i}},{\varvec{j}}}{{\varvec{c}}}_{{\varvec{i}}{\varvec{j}}}{{\varvec{I}}}_{{\varvec{i}}{\varvec{j}}}^{{{\prime}}}-{\varvec{P}}\left({\varvec{\theta}},{\varvec{s}}\right)\right)}^{2}$$

In Equation 4,

$${\left({{\varvec{c}}}_{{\varvec{i}}{\varvec{j}}}\sum_{{\varvec{i}},{\varvec{j}}}\sum_{{\varvec{k}}=0}^{{\varvec{m}}}{{2}^{{\varvec{k}}}{\varvec{q}}}_{{\varvec{k}}}^{{\varvec{i}}{\varvec{j}}}-{\varvec{P}}\left({\varvec{\theta}},{\varvec{s}}\right)\right)}^{2}$$

now reads:

$${\left(\sum_{{\varvec{i}},{\varvec{j}}}{{\varvec{c}}}_{{\varvec{i}}{\varvec{j}}}\sum_{{\varvec{k}}=0}^{{\varvec{m}}}{{2}^{{\varvec{k}}}{\varvec{q}}}_{{\varvec{k}}}^{{\varvec{i}}{\varvec{j}}}-{\varvec{P}}\left({\varvec{\theta}},{\varvec{s}}\right)\right)}^{2}$$

In Equation 5,

$$={{\varvec{c}}}_{{\varvec{i}}{\varvec{j}}}^{2}{\left(\sum_{{\varvec{i}},{\varvec{j}}}\sum_{{\varvec{k}}=0}^{{\varvec{m}}}{{2}^{{\varvec{k}}}{\varvec{q}}}_{{\varvec{k}}}^{{\varvec{i}}{\varvec{j}}}\right)}^{2}-2{\varvec{P}}\left({\varvec{\theta}},{\varvec{s}}\right)\sum_{{\varvec{i}},{\varvec{j}}}{{\varvec{c}}}_{{\varvec{i}}{\varvec{j}}}\sum_{{\varvec{k}}=0}^{{\varvec{m}}}{{2}^{{\varvec{k}}}{\varvec{q}}}_{{\varvec{k}}}^{{\varvec{i}}{\varvec{j}}}+{\left({\varvec{P}}\left({\varvec{\theta}},{\varvec{s}}\right)\right)}^{2}$$

now reads:

$$={\left(\sum_{{\varvec{i}},{\varvec{j}}}{{\varvec{c}}}_{{\varvec{i}}{\varvec{j}}}\sum_{{\varvec{k}}=0}^{{\varvec{m}}}{{2}^{{\varvec{k}}}{\varvec{q}}}_{{\varvec{k}}}^{{\varvec{i}}{\varvec{j}}}\right)}^{2}-2{\varvec{P}}\left({\varvec{\theta}},{\varvec{s}}\right)\sum_{{\varvec{i}},{\varvec{j}}}{{\varvec{c}}}_{{\varvec{i}}{\varvec{j}}}\sum_{{\varvec{k}}=0}^{{\varvec{m}}}{{2}^{{\varvec{k}}}{\varvec{q}}}_{{\varvec{k}}}^{{\varvec{i}}{\varvec{j}}}+{\left({\varvec{P}}\left({\varvec{\theta}},{\varvec{s}}\right)\right)}^{2}$$

In Equation 6,

$${\left(\sum_{i,j}\sum_{k=0}^{m}{{2}^{k}q}_{k}^{ij}\right)}^{2}=\sum_{i,j,k}{{2}^{2k}=\left({q}_{k}^{ij}\right)}^{2}+\sum_{i\le{i}^{\prime},j\le{j}^{\prime}, k\le {k}^{\prime}}{2^{k+k^{\prime}+1}}{q_k^{ij}}{q_{k^{\prime}}^{i^{\prime}j^{\prime}}}$$

now reads:

$${\left(\sum_{i,j}{c}_{ij}\sum_{k=0}^{m}{{2}^{k}q}_{k}^{ij}\right)}^{2}=\sum_{i,j}{\left({c}_{ij}\sum_{k=0}^{m}{{2}^{k}q}_{k}^{ij}\right)}^{2}+2\sum_{\begin{array}{c}i,{i}^{\prime},j,{j}^{\prime}\\ i\ne {i}^{\prime} \, or \, j\ne {j}^{\prime}\end{array}}\left({c}_{ij}\sum_{k=0}^{m}{2}^{k}{q}_{k}^{ij}\right)\left({c}_{{i}^{\prime}{j}^{\prime}}\sum_{{k}^{\prime}=0}^{m}{2}^{{k}^{\prime}}{q}_{{k}^{\prime}}^{{i}^{\prime}{j}^{\prime}}\right)$$

In Equation 7,

$$=\sum_{i,j,k}{{2}^{2k}{q}_{k}^{ij}}+\sum_{i\le{i}^{\prime},j\le{j}^{\prime}, k\le {k}^{\prime}}{2^{k+k^{\prime}+1}}{q_k^{ij}}{q_{k^{\prime}}^{i^{\prime}j^{\prime}}}$$

now reads:

$$=\sum_{i,j}\sum_{k=0}^{m}{2}^{2k}{{c}_{ij}}^{2}{q}_{k}^{ij}+\sum_{i,j}\sum_{0\le k<{k}^{\prime}\le m}{2}^{k+{k}^{\prime}+1}{{c}_{ij}}^{2}{q}_{k}^{ij}{q}_{{k}^{\prime}}^{ij}+\sum_{\begin{array}{c}i,{i}^{\prime},j,{j}^{\prime}\\ i\ne {i}^{\prime} \, or \, j\ne {j}^{\prime}\end{array}}\sum_{0\le k,{k}^{\prime}\le m}{2}^{k+{k}^{\prime}+1}{c}_{ij}{c}_{{i}^{\prime}{j}^{\prime}}{q}_{k}^{ij}{q}_{{k}^{\prime}}^{{i}^{\prime}{j}^{\prime}}$$

In addition, in the Methods section, under the subheading ‘Energy optimization algorithm for the Radon transform’,

“In Eq. 5, the second term is a linear term in the QUBO model, and the third term represents a part of the optimization value. The first term without *cij*2 is calculated as follows:”

now reads,

“In Eq. 5, the second term is a linear term in the QUBO model, and the third term represents a part of the optimization value. The first term is calculated as follows:”

And

“To derive Eq. 7 from Eq. 6, we can convert the square terms by using ${\left({q}_{k}^{ij}\right)}^{2}={q}_{k}^{ij}$ because ${q}_{k}^{ij}$ is 0 or 1. In the second term of Eq. 7, *i*, and *k* cannot be equal to *i*′,*j*′ and *k*′ at the same time. We can calculate the first term in Eq. 5 as the sum of linear and quadratic terms as in Eq. 7.”

now reads,

“To derive Eq. 7 from Eq. 6, we can convert the square terms by using ${\left({q}_{k}^{ij}\right)}^{2}={q}_{k}^{ij}$ because ${q}_{k}^{ij}$ is $0$ or $1$. In Eq. 7, $i,j$, and $k$ cannot be equal to ${i}^{\prime},{j}^{\prime}$ and ${k}^{\prime}$ at the same time. We can calculate the first term in Eq. 5 as the sum of linear and quadratic terms as in Eq. 7.”

The original Figure 1 and accompanying legend appear below.

**Figure 1:**
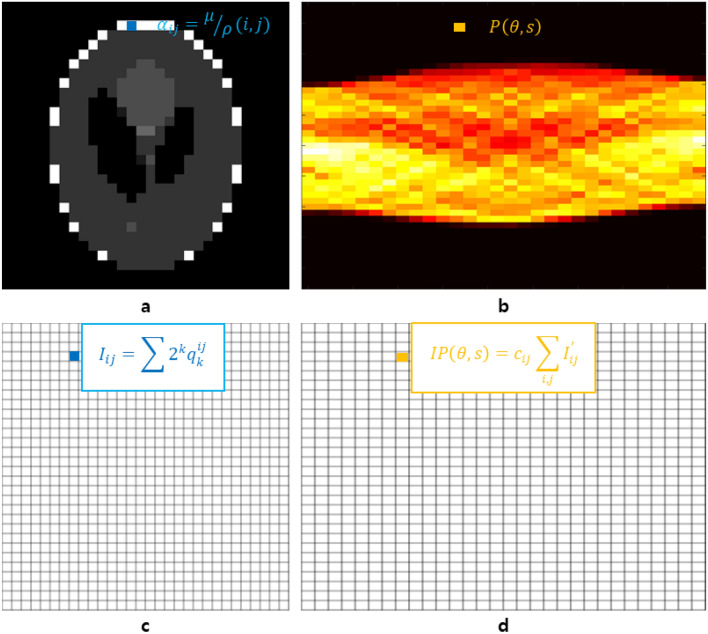
A sample, a CT image, and their two sinograms to illustrate the optimization algorithm. (**a**) This sample is a 30 × 30 Shepp–Logan phantom image. In the case of a general three-dimensional sample, it represents a cross section of the sample corresponding to its axial level. (**b**) This sinogram was obtained by using the Radon transform with the number of pixels equal to the size of the sample in (**a**). When using the data obtained from the CT system, it corresponds to the sinogram of the X-ray image. (**c**) An undetermined CT image composed of combinations of logical qubits. (**d**) The sinogram obtained by applying the projection to (**c**) in the same way as to obtain the sinogram in (**b**). In this figure, the Radon transform was applied to (**c**).

The original Article has been corrected.

