# Supplementary material for: A highly accurate quantum optimization algorithm for CT image reconstruction based on sinogram patterns
Source: Sci Rep. 2023 Sep 1;13:14407. doi: 10.1038/s41598-023-41700-6 (PMC10474150; doi:10.1038/s41598-023-41700-6)
Supplement: Supplementary file 1 — Supplementary Information. [file 41598_2023_41700_MOESM1_ESM.pdf]

In [1]:
```
import numpy as np
import matplotlib.pyplot as plt

from skimage.data import shepp_logan_phantom
from skimage.transform import rescale, radon, iradon
import cv2
```

In [2]:
```
def add_QUBO(position, values, sinogram_values):
    linear = np.square(values) # ok
    tmp1 = values*np.transpose(values) # ok
    tmp2 = np.ones(np.size(position)) - np.eye(np.size(position)) # ok
    tmp3 = linear - 2*sinogram_values*values
    return np.triu(2*np.multiply(tmp1,tmp2)+np.diag(tmp3.flatten()))
```

In [3]:
```
num_qb = 10
nx = 30
pad = 20
phantom = shepp_logan_phantom() # 400 x 400
phantom = rescale(phantom, nx/400) # 100 x 100
sol_phantom = phantom
phantom = np.pad(phantom, ((pad,pad),(pad,pad)))

dtheta = 180/nx
max_angle = 180
tnp = max_angle/dtheta
theta = np.arange(0, 180, dtheta)

rs_phantom = phantom/np.max(phantom)*(pow(2,num_qb)-1)
fn_phantom = np.round(rs_phantom, 0)  # 9 means round in Matlab
sino = radon(fn_phantom, theta)
```

In [4]:
```
plt.figure()
plt.title('Phantom Image')
plt.imshow(fn_phantom, vmin=0, vmax=pow(2, num_qb)-1)
plt.show()
sort_ph = sorted(np.unique(fn_phantom))
print(sort_ph)
```

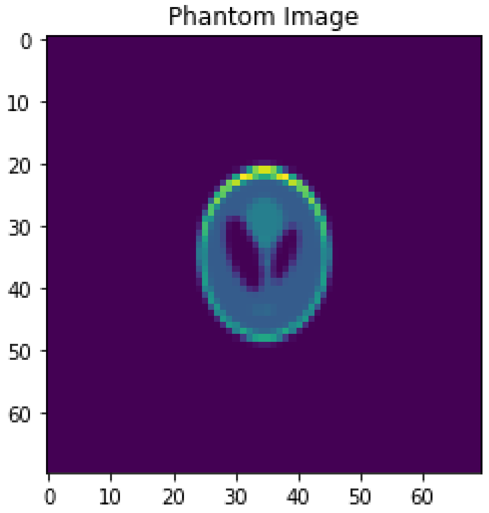


```
[0.0, 1.0, 2.0, 3.0, 4.0, 5.0, 6.0, 7.0, 8.0, 10.0, 12.0, 13.0, 14.0, 17.0, 18.0, 19.0, 21.0, 23.0, 26.0, 27.0, 28.0, 29.0, 30.0, 34.0, 38.0, 40.0, 42.0, 43.0, 47.0, 52.0, 57.0, 58.0, 64.0, 67.0, 70.0, 71.0, 72.0, 74.0, 75.0, 78.0, 81.0, 94.0, 95.0, 102.0, 107.0, 110.0, 116.0, 117.0, 123.0, 129.0, 130.0, 134.0, 138.0, 140.0, 142.0, 147.0, 155.0, 163.0, 168.0, 173.0, 176.0, 179.0, 182.0, 184.0, 193.0, 194.0, 197.0, 199.0, 200.0, 201.0, 204.0, 205.0, 207.0, 211.0, 213.0, 217.0, 218.0, 233.0, 235.0, 238.0, 239.0, 241.0, 244.0, 248.0, 262.0, 265.0, 266.0, 268.0, 270.0, 273.0, 276.0, 279.0, 280.0, 281.0, 282.0, 284.0, 285.0, 286.0, 287.0, 289.0, 291.0, 293.0, 294.0, 295.0, 296.0, 297.0, 298.0, 299.0, 300.0, 301.0, 303.0, 304.0, 305.0, 306.0, 307.0, 308.0, 312.0, 313.0, 314.0, 315.0, 316.0, 319.0, 325.0, 326.0, 327.0, 331.0, 332.0, 336.0, 337.0, 344.0, 346.0, 347.0, 356.0, 362.0, 366.0, 368.0, 371.0, 372.0, 373.0, 379.0, 401.0, 407.0, 410.0, 413.0, 414.0, 415.0, 417.0, 420.0, 424.0, 428.0, 429.0, 430.0, 435.0, 441.0, 442.0, 443.0, 452.0, 453.0, 455.0, 456.0, 466.0, 471.0, 477.0, 483.0, 507.0, 516.0, 517.0, 518.0, 524.0, 527.0, 534.0, 543.0, 544.0, 545.0, 556.0, 563.0, 567.0, 575.0, 591.0, 593.0, 604.0, 609.0, 621.0, 631.0, 677.0, 700.0, 732.0, 742.0, 743.0, 764.0, 770.0, 792.0, 797.0, 831.0, 833.0, 917.0, 954.0, 957.0, 1023.0]
```

In [5]:

```
QM = np.zeros((num_qb*nx**2,num_qb*nx**2))
Fin_Min = 0
num_row = num_qb*nx
for sino_ny in range(0,int(tnp)):
    print(sino_ny)   # 여기 ny 찍기
    temp_theta = [theta[sino_ny]]
    for sino_nx in range(0,nx+2*pad):
#         print(sino_nx)   # 여기 nx 찍기
#         if sino[sino_nx][sino_ny] == 0:
#             continue
        Arr_pos = np.array([])
        Arr_val = np.array([])
        for j in range(0,nx):
            for i in range(0,nx):
                G = np.zeros((nx+2*pad,nx+2*pad))
```

```
                G[i+pad][j+pad] = 1
                R = radon(G, temp_theta)
                for qb in range(0, num_qb):
                    Arr_pos = np.append(Arr_pos, np.array(i+num_row*(j)))
                    Arr_val = np.append(Arr_val, pow(2,qb)*np.array(R[sino_nx][0]))
        sino_val = sino[sino_nx][sino_ny]
        QM = QM + add_QUBO(Arr_pos.reshape(-1,1), Arr_val.reshape(-1,1), sino_val)
        Fin_Min -= sino_val**2
```

```
0
1
2
3
4
5
6
7
8
9
10
11
12
13
14
15
16
17
18
19
20
21
22
23
24
25
26
27
28
29
```

In [6]:

```
T = np.zeros((num_qb*nx**2,1))
for j in range(0,nx):
    for i in range(0,nx):
        Val_fn_pt = fn_phantom[i+pad][j+pad]
```

```
        for k in range(0, num_qb):
            temp_val = divmod(Val_fn_pt,2)
            Val_fn_pt = temp_val[0]
            T[i*num_qb + j*num_qb*nx + k] = temp_val[1]

tmp1 = np.matmul(np.transpose(T),QM)
tmp2 = np.matmul(tmp1,T)
print('Minimum value as our goal = ', Fin_Min)
print('By using the matrix Q, the obtained value x^TQx = ', tmp2.item())
```

```
Minimum value as our goal =  -33656657418.45889
By using the matrix Q, the obtained value x^TQx =  -33656657418.458885
```

In [7]:

```
def Q_mat(qubits,QM):
    max_d = format(len(str(qubits)), '02')

    # linear terms
    Q = {}
    for i in range(qubits):
        linear_term = format(i + 1, max_d)
        exec("Q.update({('q%s','q%s'):%s})"%(linear_term, linear_term, format(QM[i][i])))

    # quadratic terms
    for i in range(qubits-1):
        for j in range(i+1,qubits):
            if QM[i][j] != 0:
                qdrt1 = format(i + 1, max_d)
                qdrt2 = format(j + 1, max_d)
                exec("Q.update({('q%s','q%s'):%s})"%(qdrt1,qdrt2,format(QM[i][j])))
    return Q
```

In [8]:

```
from dwave.system import LeapHybridSampler
sampler = LeapHybridSampler()

qubits = num_qb*nx*nx
Q = Q_mat(qubits, QM)

answer = sampler.sample_qubo(Q)
print(answer)
```

```
  q0001 q0002 q0003 q0004 q0005 q0006 ... q9000             energy num_oc.
0     0     0     0     0     0     0 ...     0 -33653444028.65625       1
['BINARY', 1 rows, 1 samples, 9000 variables]
```

In [9]:
```
data = list(answer.first[0].values())
CT_phantom = np.zeros((nx,nx))
for j in range(0,nx):
    for i in range(0,nx):
        for k in range(0,num_qb):
            CT_phantom[i][j] = CT_phantom[i][j] + data[i*num_qb + j*num_qb*nx + k]*pow(2,k)

plt.figure()
plt.title('CT Image')
plt.imshow(CT_phantom, vmin=0, vmax=pow(2, num_qb)-1)
plt.show()
sort_ph = sorted(np.unique(CT_phantom))
print(sort_ph)
```

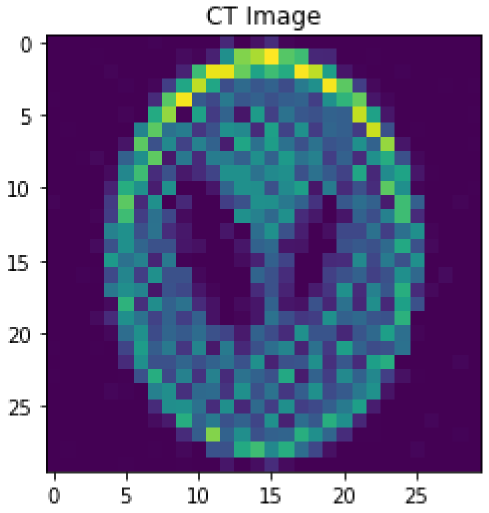

```
[0.0, 1.0, 2.0, 3.0, 4.0, 5.0, 6.0, 7.0, 8.0, 10.0, 12.0, 14.0, 15.0, 16.0, 17.0, 20.0, 21.0, 22.0, 24.0, 26.0, 27.0, 28.0, 31.0, 3
2.0, 35.0, 39.0, 41.0, 45.0, 46.0, 47.0, 48.0, 55.0, 56.0, 59.0, 63.0, 64.0, 65.0, 71.0, 72.0, 75.0, 79.0, 80.0, 84.0, 95.0, 96.0,
97.0, 99.0, 103.0, 104.0, 111.0, 112.0, 113.0, 114.0, 115.0, 118.0, 119.0, 120.0, 123.0, 126.0, 127.0, 128.0, 130.0, 131.0, 136.0,
143.0, 148.0, 151.0, 152.0, 159.0, 160.0, 162.0, 164.0, 165.0, 175.0, 176.0, 177.0, 179.0, 181.0, 184.0, 185.0, 188.0, 191.0, 192.
0, 194.0, 200.0, 202.0, 207.0, 208.0, 215.0, 216.0, 217.0, 222.0, 223.0, 224.0, 232.0, 234.0, 239.0, 244.0, 247.0, 248.0, 249.0, 25
3.0, 255.0, 256.0, 261.0, 264.0, 266.0, 267.0, 268.0, 271.0, 272.0, 278.0, 279.0, 280.0, 284.0, 287.0, 288.0, 300.0, 303.0, 304.0,
307.0, 309.0, 310.0, 311.0, 315.0, 319.0, 320.0, 321.0, 322.0, 323.0, 327.0, 329.0, 331.0, 333.0, 334.0, 335.0, 336.0, 344.0, 349.
0, 351.0, 352.0, 359.0, 360.0, 365.0, 367.0, 368.0, 370.0, 375.0, 379.0, 383.0, 384.0, 385.0, 407.0, 409.0, 411.0, 412.0, 415.0, 41
6.0, 424.0, 427.0, 430.0, 431.0, 447.0, 448.0, 449.0, 451.0, 452.0, 461.0, 463.0, 466.0, 472.0, 479.0, 480.0, 481.0, 484.0, 488.0,
495.0, 503.0, 504.0, 506.0, 511.0, 512.0, 515.0, 519.0, 548.0, 551.0, 560.0, 565.0, 570.0, 575.0, 576.0, 580.0, 581.0, 585.0, 586.
0, 592.0, 595.0, 600.0, 604.0, 606.0, 616.0, 617.0, 623.0, 624.0, 630.0, 632.0, 639.0, 640.0, 656.0, 660.0, 671.0, 703.0, 704.0, 75
2.0, 758.0, 766.0, 767.0, 768.0, 783.0, 784.0, 831.0, 835.0, 859.0, 863.0, 895.0, 920.0, 921.0, 928.0, 943.0, 991.0, 992.0, 1008.0,
1019.0, 1023.0]
```

In [10]:

```
IS_phantom = fn_phantom[pad:nx+pad,pad:nx+pad]
plt.figure()
plt.title('Image sample')
plt.imshow(IS_phantom, vmin=0, vmax=pow(2, num_qb)-1)
plt.show()
```

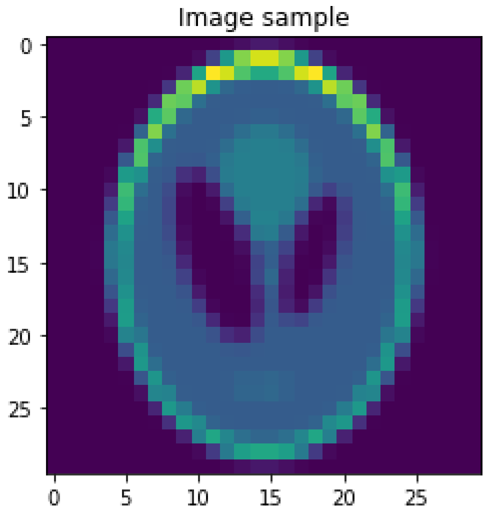

In [11]:

```
df_phantom = IS_phantom - CT_phantom
plt.figure()
plt.title('Image sample')
plt.imshow(df_phantom, vmin=-pow(2, num_qb)+1, vmax=pow(2, num_qb)-1)
plt.show()
```

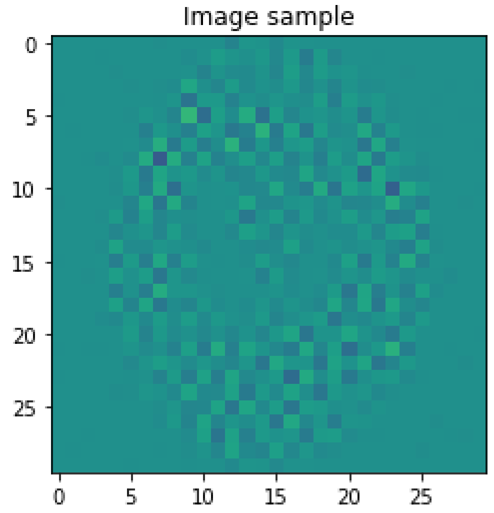
Image sample
0
5
10
15
20
25
0
5
10
15
20
25
